# Pennsylvania Hospital

**Published:** 1838-05-09

**Authors:** Henry H. Smith

**Affiliations:** Resident Surgeon


### CLINICAL REPORTS.

### PENNSYLVANIA HOSPITAL.

[*Reported by* Henry H. Smith, M. D., *resident Surgeon.*]

*Case of Amputation at the Shoulder Joint, from extensive laceration in machinery.*

Walter L——, a black man, aged 29, was admitted early on the morning of November 17th,

1837, for a very bad compound fracture of the hand, fore arm, and arm. He was engaged in attending a steam-engine, for kneading dough, at a biscuit bakery, and whilst placing the dough under a heavy lever, by which the crackers were cut, stamped, &c., his left hand was drawn into the machinery up to the shoulder. The arm was terribly lacerated, being punched at regular distances from the fingers up to the shoulder; the bones of the hand and fore arm were broken into many pieces; humerus fractured in three places; the brachial artery divided, and bare to within two inches of the axilla. He had lost much blood immediately after the injury, but had no hemorrhage at the time of his admission, one hour and a half afterwards. The skin was torn from the chest for several inches, and from the neck to within one inch of the lower jaw; the head of the humerus dislocated, and the skin torn loose some distance on the back part of the chest. On his admission, the threads holding the arm together were divided within four inches of the axilla, the vessels tied, and stump dressed with dry lint. The patient was then put to bed; ordered tinct. opii gtt. lxxx., with a little wine and water. A consultation at 12½, P. M., determined on amputation at the shoulder joint. The man having reacted sufficiently, the operation was performed by Dr. Randolph in the presence of the Surgeons of the house, and several medical men. The artery was compressed above the clavicle; and the arm being much lacerated, the vessels were first dissected up and tied; the muscles on the top were divided by the catlin; the capsular ligament divided, and the head of the bone turned out. The muscles on the axilla were next divided, the smaller vessels tied, and a flap formed by bringing the deltoid, &c., to the side. The usual dressings were applied, and the patient put to bed; pulse good; little blood lost during the operation; diet, sago; ordered t. opii gtt. lx.

*Night.* Pulse very quick—considerably over 160; cold sweat; no hemorrhage; complains of little pain; ordered wine ℥iii. in sago; laud. gtt. l., and hot tins to extremities.

*Nov.* 19th. Pulse fuller—165 in the minute; skin warm and pleasant; slept tolerably; ordered t. opii. gtt. xl., and same treatment.

*Night.* Pulse slower—about 130; skin warm and moist; laudanum repeated; continue wine, &c.

*Nov.* 19th. Great prostration; pulse quick and irregular; ordered hot egg-nogg, and stimulated very freely.

*Nov.* 20th. Died at 2, A. M., having never entirely recovered from the shock to his nervous system. There was no examination of the body.

---

*Case of Amputation of the Arm for a Compound Fracture of the Elbow Joint, ligatures removed in seven days.*

Hugh F———, aged 31 years, whilst engaged in quarrying near the city, was blown up by a charge, which exploded unexpectedly. His face, eyes, hands, arms, and legs were burnt, and the left elbow joint laid open by the ramming needle. He was seen shortly after by a physician, who removed portions of the olecranon, filled the orifice with lint, and dressed the arm with two straight splints. The next day he was brought to the city and admitted into the Hospital on the 10th of April, eighteen hours after the accident. The face was studded with particles of powder, which had penetrated the cutis; the eyes were much inflamed, and filled with sand and powder, several pieces of which were driven into the sclerotica and cornea. The left elbow joint was opened extensively, the integuments torn, the olecranon removed, and the humerus fractured obliquely above the condyles, exciting inflammation, which had extended three inches above the joint. Extremities cold, pulse slow and feeble, tongue dry and red; says he is in the habit of drinking a pint of spirits daily. The eyes were well syringed, and washed with mucilage; cloths wrung out of the same were applied to the face, with poultices of flaxseed to the burns. The elbow joint was surrounded by a warm poultice, the arm placed in a curved angular splint, extending from the shoulder to the fingers, and loosely bandaged. Ordered wine and water every two hours, and tinct. opii gtt. cxx.

*April* 11th. Has rested well; face cleansing; tongue more moist; eyes clearer; sight uninjured; arm comfortable; inflammation extending upwards.

A consultation at 4, P. M., determined on immediate amputation, which was performed by Dr. Randolph, high up, but without being able to avoid the inflamed part. On turning back the flap, the integuments were found glued to the muscles. The usual dressings were applied, and an opiate administered. Notwithstanding the unfavourable nature of the case, the arm did remarkably well; the ligatures all came away by the seventh day; the wound united very readily, and no bad symptoms followed.

*May* 2d. Three weeks after the amputation the wound is nearly entirely closed, the eyes improving, and the patient able to walk about.

---

*List of Accidents, admitted into the Pennsylvania Hospital, from April 18th to May 2d,* 1838.

One case of contusion of the shoulder, from a fall; cupped, and arm kept quiet; discharged in ten days. One case of slight contusion of the back; discharged in three days. One case of fracture of the acromion scapulæ, from the falling in of a bank of earth; dressed with clavicle apparatus. One slight lacerated wound of the hand, from a fall; dressed with adhesive plaster; afterwards poulticed, to cause suppuration and remove the dirt existing in the wound. One case of compound fracture of both bones of the leg, from an injury by the falling in of earth; dressed with fracture box and lint, wet with white of egg, so as to form an artificial scab; afterwards poulticed to promote removal of slough. One case of sprained ankle, caused by jumping from a height, dressed with fracture box, and lead water cloths; limb elevated. One case of lacerated wound of the scalp, caused by a fall from a cart; hair removed and wound united by adhesive plaster; since attacked with erysipelas and treated accordingly.

The punctured wound of the knee, reported in the last, has since been discharged, cured.

The lacerated finger was attacked by erysipelas and sloughed, the last phalanx was removed; at present healing.

